# Psychological distress experiences among patients living with HIV: a qualitative systematic review

**DOI:** 10.3389/fpsyt.2026.1788219

**Published:** 2026-06-12

**Authors:** Xiao Xu, Zhenshuai Yao, Xiaofang Zhu, Jin Wang, Shuting Tan, Pingping He

**Affiliations:** 1 Yueyang Maternal and Child Health- Care Hospital, Yueyang, China; 2 The Aging Health Research Center, School of Nursing, Medical Center, Hunan Normal University, Changsha, China

**Keywords:** experience, HIV, psychological distress, qualitative, systematic review

## Abstract

**Background:**

HIV patients endure psychological distress throughout the phases of diagnosis, treatment, and rehabilitation, which considerably affects their quality of life. A thorough comprehension of patients’ psychological distress experiences is crucial for healthcare workers to devise focused solutions for enhanced care. This study aims to consolidate evidence concerning psychological distress in HIV patients and elucidate its sources and influencing factors.

**Design:**

A systematic review and meta-synthesis of qualitative studies.

**Methods:**

A thorough literature review was performed across eight electronic databases from inception to June 2025.Qualitative studies were selected if pertinent to the psychological distress experiences of HIV patients. The quality of qualifying studies was assessed utilizing JBI qualitative research quality evaluation standard. The thematic synthesis method was utilized to amalgamate the findings.

**Results:**

A total of 1583 studies were identified through database research, and 11 studies were incorporated into the review. Three analytical themes emerged from the findings: individual factors including disease diagnosis and emotional dilemma; environmental factors including economic hardship, social environment and limited access to health support; interpersonal factors including decline in marital intimacy and insufficient support from family and friends.

**Conclusion:**

Psychological distress is a subjective phenomenon influenced by the interaction of individual, environmental, and interpersonal factors, commonly seen in HIV patients. Effective therapeutic care requires understanding psychological distress prevalence and causes. Individualized, sustainable, and adaptive psychosocial care interventions should be taken to alleviate psychological distress, improve patients’ mental health and facilitate their reintegration into society.

**Systematic Review Registration:** https://www.crd.york.ac.uk/PROSPERO/view, identifier CRD420251050269.

## 1 Introduction

By 2023, there were approximately 39 million HIV patients in globe and the incidence rate still continues to ascend in accordance with the estimation from the Joint United Nations Programme on HIV and AIDS (1). The World Health Organization has stated that HIV remains a major global public health concern (2). The advent of antiretroviral therapy (ART) in 1996 has resulted in considerable progress in HIV treatment over the last decade, markedly enhancing the life expectancy of those with HIV (3). HIV has been transformed into a manageable chronic illness; yet, individuals living with HIV encounter numerous problems that extend beyond viral management (4). Consequently, it is imperative to prioritize initiatives that enhance the quality of life for HIV patients, thereby improving their health outcomes. Multiple studies demonstrate that the quality of life for those with HIV is markedly inferior to that of the general population (5). Quality of life is affected by multiple factors, with psychological distress identified as a crucial predictor (6, 7). Psychological distress has become a notable issue for HIV patients, with research indicating that individuals may experience HIV-related psychological distress more often as the disease progresses (8).

Psychological distress encompasses a wide range of psychological phenomena, including typical negative emotional experiences such as fear, sadness, and vulnerability, as well as a typical psychological disorder like depression, anxiety, and panic, which can potentially lead to suicidal ideation (9, 10). The psychological distress experienced by HIV patients encompasses a multi-dimensional painful experience arising from the interplay of physiological, psychological, social, and spiritual factors (11). HIV patients experience a continuous and persistent psychological burden related to their condition. A cross-sectional telephone survey indicates that HIV patients are twice as likely to experience psychological distress relative to the general population (12).

With increase attention being paid to psychological distress among HIV patients, the literature concentrating on the incidence rate and risk factors of psychological distress in this group has also increased in recent years. For example, a nationwide cross-sectional household-based population survey has showed the incidence of psychological distress among 18662 HIV patients was 27.4% (13). In China, Sun et al. found the incidence rate of psychological distress among older adults living with HIV was 25.8% (14). A recent systematic review has reported the HIV-related distress prevalence in the general population ranges from 29.9% to 57.5% (15). Psychological distress is affected by several factors. Through a moderated mediation analysis, Hou et al. reported that physical activity, and positive coping significantly influenced psychological distress among 517 HIV patients (10). From a prospective cohort study conducted in 288 HIV patients, researchers found HIV-related stigma was a potential driver of psychological distress (16). Although the conclusions of these quantitative studies have demonstrated some influencing factors of psychological distress among HIV patients, considering that psychological distress is a subjective feeling, qualitative research may help better understand the psychological distress experiences, feelings and needs of patients.

Currently a growing body of qualitative studies have been conducted to focus on the psychological distress among HIV patients. For instance, West et al. conducted in-depth interviews with HIV patients and found they understood their psychological distress through culturally specific idioms (17). Additionally, Chandra et al. conducted semi-structured interviews among rural women with HIV who had multiple psychosocial vulnerabilities and reported several concerns about children, stigma, disclosure, and emotional distress when participating in an mHealth intervention (18). However, a systematic evaluation of qualitative research is still required. Several review studies have synthesized quantitative studies about the prevalence of psychological distress in HIV patients and identified various factors. The conclusions drawn from these factors are primarily shaped by the analysis of data utilizing instruments such as HIV-related psychological distress scale and Psychological Distress Scale for HIV-Infected Adolescents indicating a significant influence of the tools employed in the assessment (19, 20). In contrast, qualitative research emphasizes an in-depth and comprehensive examination of research phenomena (21). Therefore, it is essential to conduct a systematic evaluation of qualitative research regarding the psychological experiences of HIV patients.

## 2 Materials and methods

### 2.1 Aim and study design

This study seeks to analyze and integrate the genuine experiences of psychological distress among HIV patients, thereby elucidating the characteristics and determinants of this distress. A meta-synthesis was employed, as this method is widely acknowledged as the most frequently cited approach for synthesizing findings from qualitative studies. This methodology facilitates a deeper understanding of psychological distress experiences in PLWHIV during diagnosis, treatment, and other stages of the disease process. The qualitative synthesis adhered to the Jonna Briggs Institute Manual for systematic reviews of qualitative evidence (22) and was reported in accordance with the guidelines for Enhancing Transparency in Reporting the Synthesis of Qualitative Research (23).

### 2.2 Search strategy

A systematic review was performed utilizing eight databases in English and Chinese, namely PubMed, Embase, CINAHL, Web of Science, Cochrane Library, CNKI, WanFang, and Vip. A university library with extensive experience in literature research was invited to assist the research team in developing a comprehensive search strategy. The English and Chinese research were categorized into four areas: “HIV,” “psychological distress,” “experience,” and “qualitative study”. The search was restricted to articles published from March 2004 to June 2025, focusing on the period when psychological distress was defined and extensively utilized in nursing literature (24). Furthermore, researchers performed a snowball search of the references from the included studies and associated systematic reviews. Supplement file 1 outlines the specific search strategies employed for each electronic database.

### 2.3 Eligibility criteria

All the studies had to meet the criteria developed according to the PICoS framework for inclusion in this review (25) (1): participant: the participants of the studies were patients who diagnosed with HIV infection (as determined by self-reporting, self-testing, or serum tests), without restrictions on age, sex, nationality (2); phenomena of interest: authentic feelings and experiences of psychological distress among HIV patients (3);context:hospital, community, home, or other health care settings (4);study design:all types of qualitative studies such as phenomenology, grounded theory, action research, narrative research, focus interview, field notes, content analysis, thematic analysis and the qualitative part of mixed-method studies published in English and Chinese language. The exclusion criteria for studies were: (1) repeatedly published; (2) conference abstracts, protocols, and posters; (3) not available in full texts; (4) lacking complete data, with no response from the authors after contact.

### 2.4 Study selection

The results obtained from the research were exported from eight electronic databases into Endnote X9. Following the elimination of duplicate studies, two researchers independently screened titles and abstracts, subsequently conducting full-text reviews to identify eligible studies. Discrepancies between two researchers were addressed with a third researcher until consensus was reached.

### 2.5Appraisal of methodological quality

The included studies were independently evaluated for quality by two researchers utilizing the Joanna Briggs Institute critical appraisal instrument for qualitative research (26). The instrument comprises 10 items, each scored as “yes,” “no,” “unclear,” or “not applicable.” Following an independent evaluation, discussions occurred between the two researchers. In cases of inconsistent evaluation results, it is essential to consult a third researcher to achieve consensus. This review included research regardless of methodological assessment, emphasizing inclusiveness and mitigating the risk of losing valuable data. A quality appraisal was performed to critically evaluate the quality of current qualitative studies on psychological distress among HIV patients and to offer direction for future research.

### 2.6Data extraction and data synthesis

The preplanned table was utilized to extract essential information from the included studies, following the JBI General Information Extraction Form for qualitative research. This included details such as author, year of publication, country, research method, data collection method, study population, phenomenon of interest, and original findings. Furthermore, all texts, including participant quotes and the initial authors’ interpretative findings presented in the “results” or “findings” sections of each study, were copied verbatim and imported into NVivo 11 software for line-by-line coding in the thematic synthesis. Subsequent studies were categorized into established concepts, and new concepts were developed as needed.

The thematic synthesis method developed by Thomas and Harden was utilized by two reviewers to integrate the findings (27). The experience of psychological distress is a complex phenomenon that warrants exploration in studies addressing its various aspects and offering comprehensive data. Consequently, thematic synthesis was selected for this study to integrate heterogeneous studies with more comprehensive data and to develop new interpretative constructs. This method comprises three stages. The initial step involved importing the extracted data into NVivo 11 software. Qualitative descriptions, interviewee quotes, and analytical explanations from the abstract, findings, discussion, conclusion, supplementary materials, and other sections of the included studies were extracted to support the research findings. Information was coded systematically, line by line. During the second stage, codes were developed inductively to encapsulate the meaning and content of each sentence. In the third stage, studies with analogous elements were compared and contrasted, and subsequently categorized to develop descriptive themes. Descriptive and analytical themes, referred to as sub-themes and themes, were generated, leading to new interpretations of the phenomenon through this approach. Disagreements were addressed through consultation with an independent evaluator when deemed necessary. These descriptive themes were systematically reviewed and synthesized to produce analytical themes that transcended the original studies’ scope.

### 2.7 Researcher reflexivity and trustworthiness

The majority of authors are nursing academics possessing extensive experience in HIV care and prevention. Other authors possess diverse professional backgrounds, including nursing, psychology, and evidence-based nursing. Trustworthiness was established through the following methods. All researchers conducted this study rigorously following the meta-study methodology. To ensure the validity of the results, all researchers participated actively in the screening, study selection, and data synthesis processes. Throughout the research period, weekly meetings were conducted to exchange ideas and opinions, determine the research direction, and discuss findings from data analysis. The team engaged in a critical reflection on their preconceptions and assumptions regarding psychological distress to reduce the influence of bias on the findings. The findings of this study were verified by another nursing scholar with expertise in qualitative research, as well as a psychologist with relevant experience. The contents of the review were demonstrated in the manuscript.

## 3 Results

### 3.1 Literature search

**Figure 1:**
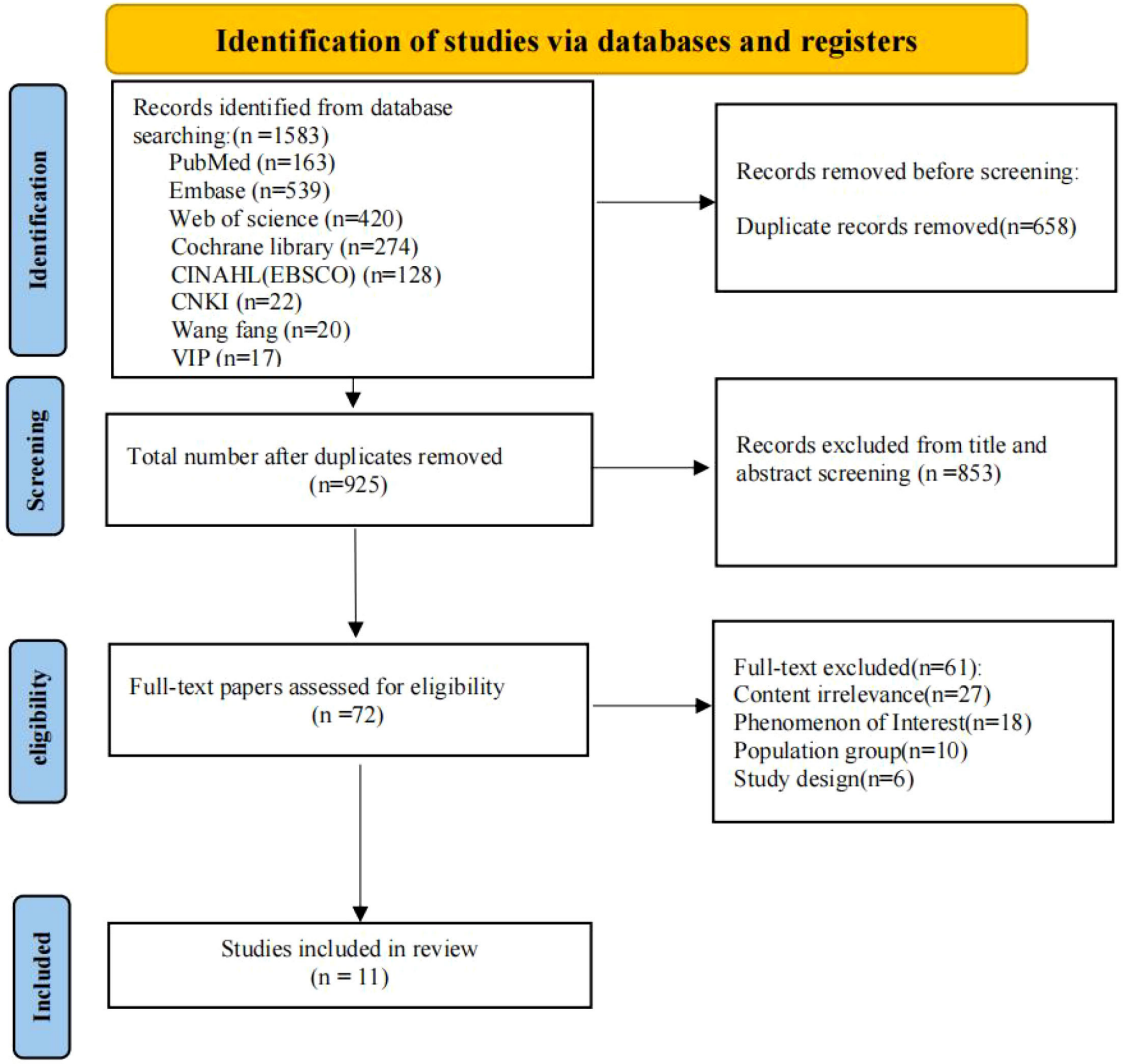
PRISMA flowchart.

A total of 1583 studies were identified through database searches. After removing duplicates and screening titles and abstracts, 72 studies were retained for full-text review. Finally, eleven studies were included in this review (17, 18, 28–36)and the PRISMA diagram is shown in **Figure 1**.

### 3.2 Study characteristics

**Table 1** summarizes the key characteristics of the studies included in this analysis. Of the eleven studies, four were conducted in China, three in the USA, two in Uganda, and one each in India and Kenyan.

**Table 1:** Summary of the included studies (N = 11).

Author(year)	Country/region	Study design	Participants	Setting	Aims	Data collection	Findings
Mo et al. (28)	China	Qualitative research design	18 college students with HIV infection	Consulting room	Having a deep understanding of the actual experience of psychological pain associated with HIV-infected college students and related factors	Semi-structured interview	Four themes: Fear of illness, Self-reproach, Perceived discrimination, Marriage constraints.
Mo et al. (29)	China	Qualitative research design	12 MSM with HIV positive	Consulting room	Gaining an in-depth understanding of the real experiences and related factors of psychological distress among MSM	Semi-structured interview	Four themes: Fear of death, Perceived discrimination, Self-blame, Economic burden
Chandra et al. (18)	India	Qualitative research design	59 rural women with HIV	telephone	Assessing the psychosocial concerns that low-literacy WLWH from rural India discussed with nurses	Semi-structured interview	Six themes: Worry about family and children, Stigma, Disclosure, Negative mood including sadness and loneliness, Intimacy Financial stress.
Kako et al. (30)	Kenyan	Qualitative research design	54 HIV-positive women	A private room	Exploring how the women perceived psychological distress and the steps they took to find support to cope with their disease.	Semi-structured interview	Four themes: Physical and emotional shock, Worry Hopelessness Suicidality.
Peltzer et al. (31)	USA	Qualitative research design	22 HIV-infected African American women	Two clinics	Examining 22 HIV-infected African American women’s experiences of psychological distress and use of coping strategies.	Semi-structured interview	Four themes: Psycho-emotional suffering Contextual factors HIV-related stigma Isolation and loneliness
Miles et al. (32)	USA	Qualitative research design	community leaders, HIV service providers, and PLWH from across the 6 counties.	community	Learning more about the distress and suffering of minority PLWH in rural areas of the South	Semi-structured interview	Seven themes: Devastation at diagnosis Fear of dying/thoughts of suicide Anger/self-blame Concerns about discrimination Living in secrecy Struggles finding health care Chronicity of distress
West et al. (17)	Uganda	Qualitative research design	20 adults living with HIV	Community	Understanding the experience and expression of psychological distress by people living with HIV	Semi-structured interview	Two themes Worry or apprehension of status Deep or many thoughts of health Prevention and treatment of disease
Feng et al. (33)	China	Qualitative research design	14 patients living with HIV over 50 years old	Outpatient department	understanding the psychological experience of elderly people living with HIV in China	Semi-structured interview	Four themes: Anxiety due to separation with their children, Fear of discrimination against their offspring, Lack of care Negative pessimistic
Payán et al (34)	USA	Qualitative research design	30 women living with HIV	clinics	Exploring the emotional, psychological, and psychosocial impacts of an HIV diagnosis on women in low-resource settings	Semi-structured interview	Three themes Feelings of depression and reduced self-worth Fear of disclosure and perceived stigma Survival Identity
Mutumba et al. (35)	Uganda	Qualitative research design	38 HIV infected adolescents	A large HIV treatment center	Identifying the psychosocial challenges and coping strategies among perinatal HIV infected adolescents in Uganda	In-depth interviews	Four themes: HIV stigma, Disclosure, Low adherence Coping strategies,
Xiao et al. (36)	China	Qualitative research design	23 patients living with AIDS	A quiet room	Investigating the psychological status and related psychosocial factors of patients living with HIV and their families	Semi-structured interview	Six themes: Development of negative emotion Strong desire to seek health information Financial burden, Uncertainty of AIDS, Discrimination, Inability to be adapted to the environment

### 3.3 Quality appraisal

All the studies met at least six standard points, with the highest number of studies meeting eight which is demonstrated in **Table 2**. None of the studies described the researcher’s situation in terms of cultural background and values, and most studies did not identify the influence of the researcher on the research.

**Table 2:** Results of the critical appraisal of the included studies.

Included studies	C1	C2	C3	C4	C5	C6	C7	C8	C9	C10	Evaluation results
Mo et al. (28)	U	Y	Y	Y	Y	N	N	Y	Y	Y	B
Mo et al. (29)	U	Y	Y	Y	Y	N	N	Y	Y	Y	B
Chandra et al. (18)	U	Y	Y	Y	Y	N	Y	Y	Y	Y	B
Kako et al. (30)	U	Y	Y	Y	Y	N	Y	Y	Y	Y	B
Peltzer et al. (31)	U	Y	Y	Y	Y	N	N	Y	Y	Y	B
Miles et al. (32)	U	Y	Y	Y	Y	N	N	Y	Y	Y	B
West et al. (17)	U	Y	Y	Y	Y	N	N	Y	Y	Y	B
Feng et al. (33)	U	Y	Y	Y	Y	N	N	Y	Y	Y	B
Payán et al. (34)	U	Y	Y	Y	Y	Y	N	Y	Y	Y	B
Xiao et al. (35)	U	Y	Y	Y	Y	Y	N	Y	Y	Y	B
Mutumba et al. (36)	U	Y	Y	Y	Y	Y	N	Y	Y	Y	B

C1. Is there congruity between the stated philosophical perspective and the research methodology? C2, Is there congruity between the research methodology and the research question objectives? C3, Is there congruity between the research methodology and the methods used to collect the data? C4, Is there congruity between the research methodology and the representation and analysis of the data? C5, Is there congruity between the research methodology and the interpretation of the results? C6, Is there statement locating the researcher culturally or theoretically? C7, Is there statement of the influence of the researcher on the research? C8, Is there representation of the participants and their voices? C9, Is there ethical approval by an appropriate body? C10, Do the conclusions drawn in the research report flow from the analysis or interpretation of the data?

Y, yes; N, no; U, unclear; NA, not applicable.

### 3.4 Synthesis of findings

#### 3.4.1 Individual factors

##### 3.4.1.1 Disease diagnosis

The diagnosis of HIV influences the probability of patients developing psychological distress. Upon receiving an HIV infection diagnosis, patients commonly experience shock and distress, frequently exhibiting intense emotional responses[“*I left that location and arrived home late. The cause was my inability to return home promptly*” *(*34)]. Patients may experience a persistent sense of sadness, which often stems from uncertainty regarding the future, insufficient information, and the challenges associated with their condition. The revelation of being HIV positive during pregnancy or postpartum was characterised as profoundly traumatic[“*At that moment, I felt extremely depressed*” (34)]. Most participants indicated that adapting to their HIV seropositive status represented the most challenging period, characterised by significant stress and uncertainty[“*When they found themselves HIV positive, they just fell down and were admitted in the ward* “ (30)]. Patients reported experiencing significant devastation and distress upon diagnosis[“*I just wanted to die. I experienced feelings of worthlessness and questioned the value of my existence. The emotional impact was profound, resulting in two weeks of crying”* (32)]. Several participants reported experiencing suicidal ideation and a sense of hopelessness after receiving their HIV-positive diagnosis[*“I felt like committing suicide. Subsequently, I purchased a rope and attempted to take my own life”* (30); *“I endeavored to run into the street to be struck by a vehicle. I made three attempts to cut my wrists (*31)].

##### 3.4.1.2 Emotional dilemma

Individuals living with HIV experience a range of emotional challenges at various stages of the disease, including post-diagnosis, throughout treatment, and during rehabilitation. In the initial phases of the disease, it disrupts normal life and induces feelings of sadness, fear, and anxiety in patients. Initially, I began taking those medications while attending boarding school[*“I experienced persistent coughing, which led to ridicule from children, resulting in feelings of distress” (*36); *“When discovering my husband’s HIV positive status was profoundly upsetting, I am genuinely astonished and surprised*” *(*31)]. Throughout the treatment process, patients frequently experience delays for examinations and treatment results, which can lead to heightened uncertainty and potentially trigger panic disorder[*“My HIV-positive status introduces uncertainty regarding my health, as individuals in this condition may experience illness at any time due to a compromised immune system” (*30)]. Additionally, several commonalities were observed in individuals with HIV experiencing psychological distress, such as feelings of hopelessness, anger, and social withdrawal[*“ Patients tended to avoid public interaction, may engage in self-talk, and often became taciturn and angry despite no external provocation”* (17)]. When receiving the mHealth intervention, patients expressed concerns regarding the future of their children[“Will I die and leave my children alone after my death? What will occur with them?” (18)].

#### 3.4.2 Environmental factors

##### 3.4.2.1 Economic hardship

For many families of individuals with HIV, supplementary medical costs represent a considerable financial strain. Financial difficulties were a persistent issue, particularly among single or widowed women [*“For children’s education, I had a lot of financial difficulties. There was no one to assist me” (*18)]. Economic issues directly contribute to the psychological distress experienced by MSM who are HIV positive, as even minor health concerns can result in complications such as pneumonia [*Consequently, I frequently required hospitalization due to pneumonia for treatment. The cost of each treatment was substantial, resulting in a significant financial burden* (28)]. Patients articulate concerns regarding job security and the process of returning to work, as evidenced by a personnel supervisor’s recommendation to remain at home for recovery from illness[*Previously, when I was in good health, I engaged in work outside the home. What are the methods to secure employment now?* (27); *“if they are employed, they worry that they will be dismissed from their job and cease working entirely”* (17)].

##### 3.4.2.2 Social environment

HIV-related stigma significantly threatens patients’ social image and adversely impacts the quality of life and mental health of people living with HIV. Multiple participants indicated instances of discrimination occurring in both educational and domestic settings[*“If anyone at school learned that my child had HIV, it would be a source of shame. If my neighbors became aware of my illness, I feared they may regard me with disdain and treat me poorly”* (18)]. The influence of diverse social and cultural contexts on psychological distress is variable. In traditional Chinese culture, HIV is associated with negative connotations, leading to discrimination against people living with HIV[*“I always feel that someone will find out that I’m a gay man and an HIV carrier. Moreover, they will engage in gossip about me in my absence, and I experience a sense of shame that inhibits my ability to confront others’*’ (27)].

##### 3.4.2.3 Limited access to health support

A direct cause of psychological distress in HIV patients occurs when healthcare providers fail to promptly address their needs. Rural towns exhibited a scarcity of mental health services, which were primarily viewed as accessible only to individuals with severe mental illness or suicidal idea[*“We had a mental health center but unless it was related to alcohol, drugs, or you express the intention of committing suicide” (*32)]. The absence of health insurance, restricted access to healthcare in rural areas, and the considerable distances to HIV clinics in tertiary care facilities collectively posed significant challenges in obtaining healthcare[*“Many patients must apply for state disability benefits to obtain funding for medications, this process was complex and time-consuming”* (32)].

#### 3.4.3 Interpersonal factors

##### 3.4.3.1 Decline in marital intimacy

This disease presents numerous obstacles for patients in their romantic relationships and marriages. Most HIV patients feel distressed because their sexual relationships are restricted when they have not been married[*“Now I was at a loss as to what to do when I encountered a partner I like, I feared that disclosing my illness would result in his departure” (*28)]. The absence of sexual activity contributes to a decrease in marital intimacy and can lead to the deterioration of the marital relationship after marriage[*“My husband does not care for me and becomes indifferent to my feelings. He had married another woman” (*18); *“The issues you may encounter include verbal abuse from your husband and subsequent mistreatment”* (17)].

##### 3.4.3.2 Insufficient support from family and friends

Family and friends serve as essential sources of social support for individuals living with HIV. Family members aware of the diagnosis did not consistently provide support, as indicated by their tendencies to avoid, ostracize, or blame the person living with HIV. The respondents who were cognizant of their illness experienced neglect from their children and were even forcibly excluded from their homes[“*After disclosing the truth, they ceased requesting financial assistance, and their father subsequently asked me to relocate and live independently*” *(*33)]. A significant number of respondents indicated challenges in disclosing their HIV status to peers and sexual partners, citing potential loss of friendships and increased stigmatization [“It is very hard to tell people you are positive, you may even lose your friends “;”I do not want my friends to pity me and gossip about me” (36)].

## 4 Discussion

This study provides a comprehensive summary of the psychological distress experienced by HIV patients across eleven studies. During the diagnosis, treatment, and rehabilitation phases, individuals living with HIV encounter varying degrees of psychological distress, with distinct underlying causes for this distress. Distress can originate from individual factors, environmental influences, or interpersonal skills. By integrating findings from existing qualitative studies, this qualitative meta-synthesis comprehensively examined the psychological experiences of HIV patients, spanning from the point of diagnosis throughout the course of a chronic illness. This contribution lays a solid scientific groundwork for the future development and implementation of HIV mental health practice.

### 4.1 Individual factors are the primary causes of psychological distress

Psychological distress is a prevalent symptom in people living with HIV and represents a significant challenge. The psychological dilemmas faced by HIV patients from diagnosis to rehabilitation vary across different stages of the disease (37). Upon receiving a new HIV diagnosis, patients confront the associated psychological distress (38). The diagnosis and treatment of HIV disrupts life patterns and significantly adversely affects individual appearance and functionality (39). Community discourse around visible physical changes associated with HIV remains common, reinforcing the perception that HIV is a lethal and incurable disease (40). A cross-sectional descriptive study showed that patients undergoing treatment displayed lower psychological adaption and coping capacities, resulting in a perceived loss of life and the likelihood of psychological distress (41). Furthermore, a notable decline in the quality of life for individuals living with HIV is a significant challenge, as many must contend with both the physical effects of the virus and the compounded pressures of psychological stressors (42). For instance, anxiety and depression are prevalent mental health issues globally, particularly among individuals living with HIV, with average prevalence rates of 15.5% for anxiety and 18.1% for depression (43, 44). This indicates the importance of examining the dynamic changes in psychological distress among HIV patients across various treatment stages and identifying the factors influencing psychological distress at these stages. Improving the comprehension of these factors including depression, anxiety and stigma can guide the design of intervention measures and identify the most effective trajectory for these measures. The integration of mental health care within HIV care and treatment settings enhances the provision of comprehensive care (45). Moreover, a meta-analysis showed nurse-led self-care interventions effectively improve the quality of life and reduce psychological distress in people living with HIV (46). A possible explanation is that nurse-led interventions focus on education and skill development, enabling individuals living with HIV to gain the knowledge and techniques necessary for effective health management. This enhancement in capability increases self-efficacy, leading to greater confidence in managing the physical and mental challenges associated with HIV, thus effectively reducing depressive symptoms (47). Additionally, nurses play a crucial role in self-care interventions by providing personalized care and psychological support (48). Nurses typically exhibit strong communication skills and empathy, which facilitate the establishment of trusting relationships with patients (49). This trust can minimize risk factors for depression, such as loneliness and helplessness, thereby reducing psychological distress.

### 4.2 Environment constraints are external factors of psychological distress

Adverse environmental factors diminish typical social participation, resulting in psychological distress. Expenditures on treatment costs, along with a reduction in labor capacity during treatment and rehabilitation, result in lower income (50). This imposes a significant economic burden on individuals living with HIV and has lasting effects on their physical, psychological, and social well-being. Medical personnel can perform screening and offer support information and financial assistance to economically disadvantaged patients with HIV infection (51). Relevant departments should concurrently facilitate employment opportunities for PLWHIV and enhance financial support for AIDS organizations (52). Government agencies, including labor and social security departments, health departments, and civil affairs departments, along with non-governmental organizations, charitable organisations, and socially responsible enterprises, can offer opportunities for people living with HIV to re-enter the workforce. Support can be provided through multiple avenues, such as policy initiatives, employment services, vocational training, and financial assistance (53). Furthermore, family formation is a significant aspect of Chinese culture, shaped by sociocultural characteristics, traditions, and religious beliefs. The diversification of HIV treatment methods, improvements in survival rates, and the introduction of the three-child policy have influenced many patients’ perceptions of traditional fertility concepts, resulting in a positive attitude towards fertility demand (54). It has been reported that approximately 47.8% of people living with HIV have a desire to have a child (55). Consequently, the prompt recognition of patient fertility issues is essential. Concerns regarding fertility surpass the psychological pressure and adverse emotions associated with HIV among women of childbearing age (56). Medical professionals must prioritize patients’ reproductive needs, implement timely treatment interventions, offer professional informational support, and optimize patient fertility to effectively reduce the prevalence of disease among young and middle-aged individuals living with HIV.

### 4.3 Interpersonal support are critical factors of psychological distress

This research demonstrates that interpersonal factors constitute significant risk factors for psychological distress in individuals living with HIV. Alterations in sexual health and function during and after treatment may significantly influence interpersonal and intimate relationships among individuals living with HIV (57). The spouse of a patient experiences concurrent stress due to life changes and psychological adaptability, which subsequently reduces marital intimacy (58). Advocating for binary intervention strategies targeting couples is essential for the enhancement of intimate relationships (59). These strategies can enhance the psychological well-being and social adjustment of both partners, thus reducing their psychological distress. The failure to create or maintain a strong family and social network is a contributing factor to the emergence of personality disorders. Interpersonal support effectively reduces psychological stress in challenging situations and improves the physical and mental health of individuals with HIV (60). This study found that understanding and support from spouses, family members, and friends are critical in preventing psychological distress. Individuals living with HIV demonstrate heightened psychological sensitivity during the course of their illness. It is advisable to maintain regular communication. It is important for friends and family members to avoid intentionally reducing interactions with patients under the guise of protective intentions. This includes refraining from avoiding discussions that may increase patients’ anxieties, frequently enquiring about their condition in a way that may exacerbate fears, or assuming that patients need more rest (61). Such behaviors may adversely affect their condition. Future intervention research should focus on the interplay among family, hospital, and community, aiming to strengthen the social network of HIV patients and ensure they obtain adequate social resources and support. Inadequate support from other social sources represents a significant risk factor for psychological distress in HIV patients. Peer support offers a reciprocal advantage with considerable ramifications for public health systems, clinical healthcare providers, individuals with HIV, and HIV peer volunteers (62). It is essential to create efficient models for HIV peer support practice. Collaborative initiatives across pertinent departments and personnel, in conjunction with HIV peer volunteers, should be implemented to develop support measures (63). Moreover, initiatives must be implemented to identify and direct individuals with HIV into the healthcare system, ensuring thorough treatment and continuity of care (64). These approaches seek to increase the mental health of HIV patients, improve their quality of life, and promote the normalization of HIV.

### 4.4 Limitations

This study has some limitations. First, none of the studies that were included explained how the researcher affected the study or how the study affected the researcher. Additionally, the researcher’s context from the standpoint of cultural background and values is not well explained, which could lower the overall trustworthiness of the research findings. Second, due to language restrictions, only English and Chinese researches were included, potentially introducing selection reporting bias. Last but not least, psychological distress evolves together with the patient’s health trajectory, from the onset of early symptoms to diagnosis, treatment, and survival to the end of life. Nevertheless, the qualitative research was carried out at a single point in time in the majority of the studies, which might not provide a comprehensive picture of psychological distress.

### 4.5 Implications for practice and research

Patients encounter psychological distress at various stages of HIV infection. The causes of psychological distress vary across different stages. Medical personnel ought to apply specific interventions for psychological distress that are informed by the factors pertinent to various stages. During the initial diagnosis phase, a significant emotional response to the HIV diagnosis is common. Strategic disclosure of unfavorable information may facilitate acceptance among individuals living with HIV. This approach can mitigate patients’ levels of psychological distress. Medical staff should assess the preferences of patients and their families prior to disclosing unfavorable news concerning an HIV diagnosis. Choosing an appropriate notification model based on preference is advisable. Medical staff must judiciously decide whether to inform the patient or their family members, ascertain the information to be disclosed, and implement a gradual approach to facilitate the acceptance of adverse news. The treatment phase is characterised by prolonged physical and mental discomfort resulting from treatment side effects. The duration of treatment is a primary concern for medical professionals. Special attention should be directed towards the psychological distress associated with fertility issues in patients of childbearing age, as this represents a distinct form of distress encountered by individuals in this demographic. It is crucial to consider individual, therapeutic, environmental, and interpersonal factors in the development of intervention measures. Enhancing support for patients from family, friends, and healthcare professionals is crucial, as is fostering effective communication between patients and their networks and aiding in the alleviation of psychological distress in patients.

## 5 Conclusion

This systematic review synthesized the experiences of psychological distress among HIV patients, with the objective of enhancing medical staff’s attention to the mental health of these patients. Understanding the prevalence of psychological distress and its contributing factors is essential for effective clinical management. Psychological distress is a subjective experience shaped by the interplay of individual, environmental, and interpersonal factors, frequently observed in HIV patients. To enhance management, the characteristics and external environment of HIV patients must be meticulously considered during the provision of routine nursing care. Targeted intervention plans are essential for effectively reducing the incidence of psychological distress, which is vital for restoring patients’ mental health and facilitating their reintegration into society.

## Data Availability

The original contributions presented in the study are included in the article/supplementary material, further inquiries can be directed to the corresponding author/s.
